# Metabolomic Response throughout 16 Weeks of Combined Aerobic and Resistance Exercise Training in Older Women with Metabolic Syndrome

**DOI:** 10.3390/metabo12111041

**Published:** 2022-10-30

**Authors:** Amanda V. Sardeli, Alex Castro, Victor B. Gadelha, Wellington M. dos Santos, Janet M. Lord, Cláudia R. Cavaglieri, Mara Patrícia T. Chacon-Mikahil

**Affiliations:** 1 Laboratory of Exercise Physiology, School of Physical Education, State University of Campinas, Campinas 13083-851, Brazil; 2 Gerontology Program, School of Medical Sciences, State University of Campinas, Campinas 13083-970, Brazil; 3 Institute of Inflammation and Ageing, University of Birmingham, Birmingham B15 2TT, UK; 4 Nuclear Magnetic Resonance Laboratory, Department of Chemistry, Federal University of São Carlos (UFSCar), São Carlos 13565-905, Brazil

**Keywords:** combined training, metabolomics, elderly, exercise, physical training, metabolites, metabolism, aging, cardiovascular health, menopause

## Abstract

Increases in longevity and obesity have led to a higher prevalence of Metabolic Syndrome (MetS) and several chronic conditions, such as hypertension. The prevalence of MetS and hypertension increases with advancing age and their detrimental effects on health can be attenuated by physical activity. Combined aerobic and resistance exercise training (CT) is recommended to maintain good health in older adults and is known to generate important metabolic adaptations. In this study we performed a metabolomics analysis, based on Hydrogen Nuclear Magnetic Resonance (1H NMR), to investigate the kinetics of changes in metabolism in non-physically active older women with MetS in response to 16 weeks of CT. A subset of women with MetS were selected from a larger randomized trial (that included men and women without MetS), with 12 participants on CT and 13 from the Control Group (CG). CT comprised walking/running at 63% of VO_2_max, three times/week, and resistance training (RT), consisting of 15 repetitions of seven exercises at moderate intensity, twice/week. Serum metabolomic profile was analysed at baseline (0W), 4 (4W), 8 (8W), 12 (12W) and 16 weeks (16W) for CT or CG. Cardiorespiratory fitness, RT load, blood pressure, body composition, lipid and glycaemic profile were also assessed. After 16 weeks CT increased cardiorespiratory fitness (13.1%, *p* < 0.05) and RT load (from 48% in the lat pulldown to 160% in the leg press, *p* < 0.05), but there were no changes in MetS parameters, such as body composition (Body Mass, Body Mass Index (BMI), body fat percentage and waist circumference), blood pressure, lipid and glycaemic profile. However, we identified potential higher substrate to the tricarboxylic acid cycle (increase in 2-Oxobutyrate from 0W (0.0029 ± 0.0009) to 4W (0.0038 ± 0.0011) and 8W (0.0041 ± 0.0015), *p* < 0.05), followed by alterations (different from 0W, *p* < 0.05) in the production of ketone bodies (3-Hydroxybutyrate, 0W (0.0717 ± 0.0377) to 16W (0.0397 ± 0.0331), and Acetoacetate, 0W (0.0441 ± 0.0240) to 16W (0.0239 ± 0.0141)), which together might explain the known improvement in fatty acid oxidation with exercise. There was also a late increase in ornithine at 16W of CT. Further studies are needed to investigate the association between these metabolic pathways and clinical outcomes in this population.

## 1. Introduction

Increases in longevity and obesity globally [1,2] have boosted the prevalence of Metabolic Syndrome (MetS) and several chronic conditions, such as hypertension [3,4,5,6,7]. MetS is defined by the presence of a combination of risk factors, such as abdominal obesity, atherogenic dyslipidemia (elevated triglyceride, small LDL particles, low HDL cholesterol), raised blood pressure and insulin resistance (with or without glucose intolerance) [8]. The prevalence of MetS and hypertension increases with age and the harmful effects of both conditions can be attenuated by physical activity. For example, exercise training has been shown to reduce metabolic dysfunction and treat the associated risks in individuals with MetS [8]. Exactly how these benefits are achieved is still not fully understood.

Currently, metabolomic approaches have been used to test the effect of exercise interventions on metabolic profiles of healthy and diseased populations [9,10,11,12]). Metabolomics simultaneously measure numerous metabolites, which in total reflect the downstream outcomes of cellular metabolism, termed the metabolome, facilitating the study of metabolic networks and pathways [13,14]. The main techniques used to measure metabolites in body fluids are mass spectroscopy and nuclear magnetic resonance (NMR) spectroscopy. NMR has several advantages as an analytical platform for metabolomics; notably, it is highly reproducible, allowing inter-laboratory comparisons and provides highly accurate quantitative measurement of a broad range of metabolites with the use of a single internal standard. NMR also does not destroy the sample, allowing multiple replications of experiments. As a result, 1H NMR spectroscopy is one of the most widely employed metabolomic techniques for detecting and measuring metabolites related to physical activity, exercise and health [15,16]. In this sense, several studies have addressed associations between changes in metabolomic profile and changes in other clinical outcomes after exercise training in healthy, obese and older adults [9,10,11,12,17]. For example, Duft et al. [9] found tyrosine was associated to higher strength and VO_2_max, while glutamine and pyruvate were associated with lower fat and higher lean mass; glucose and creatine were associated with higher strength and lower waist circumference post 24 weeks of combined training in obese men. The comprehensive metabolic profiling possible with metabolomics analysis allows the exploration of complex interactions that lead to exercise training adaptations. Even different types of exercise training interventions can lead to similar gains in cardiorespiratory fitness associated with the regulation of common pathways involving aminoacyl-tRNA biosynthesis, carbohydrate and amino acid metabolism [10].

Since MetS components and associated cardiovascular risks differ between sexes, the potential targeting of exercise therapies based on sex deserves further investigation, including the increased representation of women in clinical trials [18]. Any sex-related differences could be related to differences in body composition, with adiposity and lean muscle mass differences able to influence the physiological and metabolic responses to exercise [19,20].

The aim of the present study was to test the effects of 16 weeks of combined aerobic and resistance exercise training on metabolic profile and MetS components in hypertensive older women with MetS. The use of metabolomics to quantify metabolites will allow the characterization of candidate biomarkers, which would be potentially used as diagnostic tools for disease screening, prognosis and targeting of therapeutic interventions, including exercise [21,22].

## 2. Materials and Methods

### 2.1. Participants

All participants provided written, informed consent approved by the Ethics Committee. The protocol was registered on the Brazilian registry of clinical trials ((https://ensaiosclinicos.gov.br/rg/RBR-3yxds4, accessed on 20 September 2022), identifier [U1111-1181-4455]). Television and radio advertisements invited the potential participants to contact the researchers. To be included in the study the participants had to be older than 60 years, not be physically active (frequency of physical activity less than two sessions per week); have not regularly participated in any training program in the previous 6 months; have controlled hypertension; and be approved for involvement after a clinical assessment (cardiology and clinical ergometry). In addition, participants had to confirm they did not have any other complications that may be risk factors for the practice of exercise, such as obesity greater than grade 2 (body mass index [BMI] greater than 35), coronary artery disease, Diabetes Mellitus, any chronic lung disease, limiting osteoarticular disease, peripheral vascular disease, smoking or use of any medication that may interfere with the physiological responses to beta-blocker and/or insulin tests. In the main trial (where men and women without MetS were included) we randomised 52 participants [23] for one of the two experimental groups: Control Group (CG) or Combined Training (CT). The participants in each group were balanced by sex, age, blood pressure (BP) and BMI. All analyses and training sessions were carried out on the Campus of the School of Physical Education at UNICAMP. For the present sub-study, we selected the subset of women with MetS (3 out of the following 5 criteria: waist circumference > 88 cm; SBP > 130 mmHg or DBP > 85 mmHg or anti-hypertensive medication; blood glucose > 110 mmHg; Tryglicerides > 150 mg/d; and HDL < 50 mg/dL [24]) for analysis, leading to 12 participants in the CT and 13 participants in the CG. 

We considered the moderate effect sizes (ƒ = 0.3) observed in previous studies with a similar design [9] to calculate the necessary sample size to ensure at least 80% statistical power (1-β) in the analyses, assuming a type I error rate (α) of 5% [25].

### 2.2. Experimental Protocol

Blood samples to assess metabolomic profile and resistance training (RT) load progression were taken at baseline (0W), 4 (4W), 8 (8W), 12 (12W) and 16 weeks (16W) of CT and CG. Both groups were advised to maintain the same diet across the intervention period. VO_2_max was assessed at 0W, 8W and 16W and all other variables were assessed at 0W and 16W. Participants were scheduled for assessment at the same time of day and with the same evaluator for all time points. At least 48 h rest occurred between the prior training session and the assessments, and the participants were asked to fast for 12 h before the blood draw, cardiorespiratory fitness, and blood pressure assessments. If the participants had forgotten to take their standard medication or underwent any transitory health issue, the monthly assessments were rescheduled for the following week. Participants, training instructors and evaluators were not blinded for data collection; however, the overall data exported for analysis were blinded.

### 2.3. Body Composition

Body composition assessments were performed before and after intervention using a full-body plethysmograph (BOD POD^®^; Body Composition System; Life Measurement Instruments; Concord, CA, USA). In all evaluations, the ambient temperature and humidity conditions were maintained between 21–23 °C and ~60%, respectively, without significant variations in atmospheric pressure, according to the manufacturer’s recommendations [26]. From these data, the body density was converted into a percentage of fat using the Siri equation [27]. Height and weight were assessed to determine BMI and waist circumference was also recorded.

### 2.4. Cardiorespiratory Fitness

The participants performed a test on a treadmill (Quinton TM55. Bothell, WA, USA), where gas exchanges were collected continuously, breath by breath, through a metabolic gas analysis system (CPX, Medical Graphics, St. Paul, MN, USA). The protocol started with a warm-up speed of 4 km·h^−1^ for 2 min, followed by increments of 0.3 km·h^−1^ every 30 s until exhaustion. Then, a 4-min recovery period was performed, with the first minute at 5 km·h^−1^, decreasing 1 km·h^−1^ every minute [28,29]. The participants were verbally encouraged to reach maximum effort speed, especially when close to exhaustion. The criteria to confirm maximum effort, were a respiratory exchange ratio greater than 1.15 (Respiratory Exchange Ratio—RER), heart rate between 10 bpm of the maximum predicted by age and rate of perceived exertion (RPE) above 18 on a 20-point scale [30]. The VO_2_max is the point where the plateau of oxygen consumption occurs even when the intensity of effort increases [28]. In case of absence of a plateau, which is common in tests of sedentary or sick patients, the use of VO_2_ peak can be assumed as the VO_2_max [31]. The VO_2_ peak was extracted from the highest average of the final 30 s of the oxygen consumption values [32]. Furthermore, to guarantee maximum values, by reducing the familiarization effects in the results, a re-test was performed before the beginning of the intervention and the higher of the two values was used for analysis.

### 2.5. Blood Pressure

After remaining at rest, in a supine position for 15 min, BP was measured by digital photoplethysmography, using the Finometer Pro^®^ (Finapres Medical System, Amsterdam, The Netherlands). The cuff was positioned on the distal phalanx of the middle finger of the right arm, calculating the mean 300 beats in a rest period [33,34,35].

### 2.6. Blood Measures

Blood draws were obtained from an antecubital vein after a 12 h fast and each participant had the blood collected always at the same time repeated every 4 weeks during the 16-week intervention. For metabolomic analysis, the blood samples were centrifuged at 2655× *g* for 10 min and then the serum aliquots were stored at −80 °C until analysis. Plasma glycemia and lipid profile analyses were performed by standardized methods in a routine clinical laboratory. Lipids measured were total triglycerides (TG) and total cholesterol (TC) by enzyme-trinder method, HDL by selective detergent method and LDL by Friedewald equation (LDL = (TC − HDL) − (TG/5)). C-reactive protein (CRP) was assessed by multiplex assay and tumour necrosis factor alpha (TNF-α) was assessed using enzyme-linked immunosorbent assay (ELISA) and both methods using commercial kits (R&D Systems, Minneapolis, MN, USA).

### 2.7. Blood Sample Preparation for Metabolomics Analysis

Three kDa filters (Amicon Ultra, Merck KGaA, Darmstadt, Germany) were washed with 500 μL of Milli-Q water, followed by centrifugation at 14,000 rcf for 10 min at 4 °C. This process was repeated five times. After the fifth wash, filters were reversed and rotated at 8000 rpm for 5 s to eliminate any residue of Milli-Q water. Serum (500 μL) was then added to the filter, which was centrifuged at 14,000 rcf for 45 min at 4 °C. 250 μL of filtered serum was then transferred to a 5 mm NMR tube (Wilmad Standard Series 5 mm, Sigma-Aldrich, Gillingham, UK) containing 60 μL of phosphate buffer ((monobasic sodium phosphate, NaH_2_PO_4_ H_2_O, 137.99 g·mol^−1^; dibasic sodium phosphate, Na2HPO3, 141.96 g·mol^−1^), TMSP-d4 (3-(trimethylsilyl)-2,2′,3,3′-tetradeuteropropionic acid), at 5 mmol·L^−1^ in D2O (6.06 μL) (internal reference)) and 290 μL of D2O (99.9%; Cambridge Isotope Laboratories Inc., Tewksbury, MA, USA)

### 2.8. NMR Spectrum Acquisition and Metabolite Quantification

Metabolomic analysis was performed in the National Biosciences Laboratory (LNBio—http://lnbio.cnpem.br/, accessed on 20 September 2022) at the Energy and Materials Research Center in Campinas, SP, Brazil. Each spectrum of 1H NMR was acquired using VnmrJ software (Varian NMR Systems, Palo Alto, CA, USA) and a Varian Inova 1H NMR spectrometer (Agilent Technologies Inc., Santa Clara, CA, USA), operating at 600 MHz frequency and a constant temperature of 298 K (25 °C) [36]. A total of 256 free induction decays (FID) were collected over a spectral width of 8000 Hz, with an acquisition time of 4 s and relaxation delay intervals of 1.5 s. 

After acquisition, the spectral phase and baseline corrections, as well as the characterization and quantification of the metabolites present in the serum samples, were performed using the Suite Professional 8.31 Chenomx NMR software (Chenomx Inc., Edmonton, AB, Canada), using the TSPd4 signal (known concentration) as an internal reference for quantifying the concentrations of other metabolites. All spectra were processed with 0.3 Hz line broadening (lb) to improve the signal/noise ratio. The samples were randomly profiled blindly in relation to the evaluator to avoid external interferences and subjectivity, thus preventing the biasing of the analyses and results. The spectra were adjusted, and the water signal region of the spectrum was deleted, as it would interfere with the quantification of metabolites.

### 2.9. Additional Assessments

The monitoring of infections was carried out through a questionnaire [28]. When positive changes in infection were observed, the date of assessment was postponed. Participants’ pre- and post-intervention diet reports were recorded and the absence of change in their habits were confirmed in a previous study, encompassing the total men and women study cohort [37]. For all evaluations and tests, the same evaluator performed the assessments to avoid bias.

### 2.10. Exercise Training Protocol

We applied a 16-week combined training (resistance and aerobic exercise) program, based on the American College of Sports Medicine (ACSM) guidelines for older adults [38]. The combined training had a frequency of three sessions per week, two sessions consisting of seven resistance exercises, and three sessions of 50 min of aerobic exercise on the treadmill. Two of these sessions were combined, beginning with resistance training (RT) and ending with aerobic training (AT), and one session had only AT. The RT consisted of the following exercises: knee extension in the extensor chair, knee flexion in the flexor chair, horizontal leg press, bench press with barbell lat pulldown, plantar flexion standing on the step and abdominal supra on the mat. One set was performed for each exercise containing 15 repetitions. There was a one-minute interval between exercises. The RT load was prescribed using rate of perceived exertion (RPE) in each exercise. The load was increased every time the participants reported RPE values below 7 on the scale ranging from 0 to 10 [38,39]. The AT consisted of 50 min of walking or running on a treadmill, depending on the level of physical conditioning of each participant, who were instructed to maintain the pace to achieve 63% maximum oxygen volume (VO_2_max), which is equivalent to 60% of the reserve VO_2_ [38,40,41].

### 2.11. Statistical Analysis

The normality of data was tested by the Shapiro–Wilk test. When any of the data groups of each variable did not present a normal distribution (*p* ≤ 0.05), the entire dataset was submitted to natural logarithmic transformation. After analysis, data were presented in their original scale for an easier interpretation. When the data were normal, the raw data group was analysed. Baseline characteristics used for randomization were compared between groups using independent *T*-test. A repeated measures analysis of variance (analysis of variance) was used to compare the progression of training loads (load used in strength exercises) throughout the sessions only in the CT group.

Multivariate statistical analyses were performed to analyse the metabolites, such as principal component analysis (PCA) and partial least squares discriminant analysis (PLS-DA). We performed these analyses via the online MetaboAnalyst 3.0 software. Logarithmic transformation (Log10) was also performed for the metabolite values for greater symmetry between the data distribution [42]. PCA analyses were performed in order to examine the differentiation in the global metabolic profile between the CT and CG groups. The PLS-DA analyses were used to identify discriminant metabolites that could explain the changes in the metabolic profile between the groups. 

Afterwards, univariate analyses were performed using the SPSS software (version 24). Mixed linear model analyses (MMA) were used for all variables of the metabolomic analysis, considering the five moments (0W, 4W, 8W, 12W and 16W) and the two groups (CT and CG) as fixed effects, and subjects as random effects. Cardiorespiratory fitness was analysed by MMA only at three timepoints (0W, 8W and 16W). For the different points, group and timepoint × group interactions in all MMA, fixed effects were considered, using the first-order autoregressive covariance model (AR1). Sidak’s post hoc test was used when there was significant timepoint × group interaction. 

## 3. Results

To be included in the analysis the participants had a minimum attendance of 85% in training sessions, and all of them achieved this target. Characteristics including body composition, blood pressure and lipid and glycaemic profile of the participants are shown in Table 1. There was no statistical difference between groups or between 0W and 16W for all variables presented on Table 1.

### 3.1. Cardiorespiratory Fitness and RT Load

CT increased VO_2_ peak from 21.06 ± 3.09 mL·kg^−1^·min^−1^ at 0W to 23.46 ± 3.19 mL·kg^−1^·min^−1^ at 8W (+11.4%, *p* = 0.03) and maintained the higher level at 16W (23.82 ± 3.2 mL·kg^−1^·min^−1^, 13.1%, *p* = 0.03), while CG did not change VO_2_ peak from 0W (19.97 ± 4.45 mL·kg^−1^·min^−1^) to 8W (21.35 ± 6.21 mL·kg^−1^·min^−1^, *p* = 0.59) or to 16W (20.88 ± 5.77 mL·kg^−1^·min^−1^, *p* = 0.82). The RT load underwent a continuous progression and some measures reached statistical significance (Table 2). A significant increase was seen in the load of the lat pulldown from 0W to 8W (*p* = 0.01), which was maintained until 16W (8W vs. 16W, *p* = 1.0 and 0W vs. 16W *p* < 0.001); the increased bench press from 0W to 8W (*p* < 0.001); the leg extension from 0W to 4W (*p* = 0.006); the leg flexion from 0W to at 8W (*p* < 0.001); and the leg press from 0W to 8W (*p* < 0.001).

### 3.2. Metabolomic Response to Exercise

**Figure 1:**
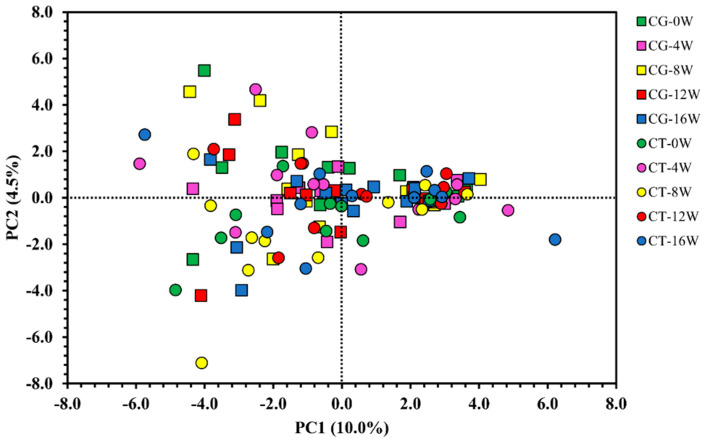
Principal component analysis (PCA). Control Group (CG) is represented by the square (☐) and the Combined Training (CT) is represented by the circle (◯). Colours vary according to the time of data collection; 0W = green: pre-training; 4W = pink: 4 weeks; 8W = yellow: 8 weeks; 12W = red: 12 weeks; 16W = blue: 16 weeks. The present model used data for all 66 metabolites detected.

**Figure 2:**
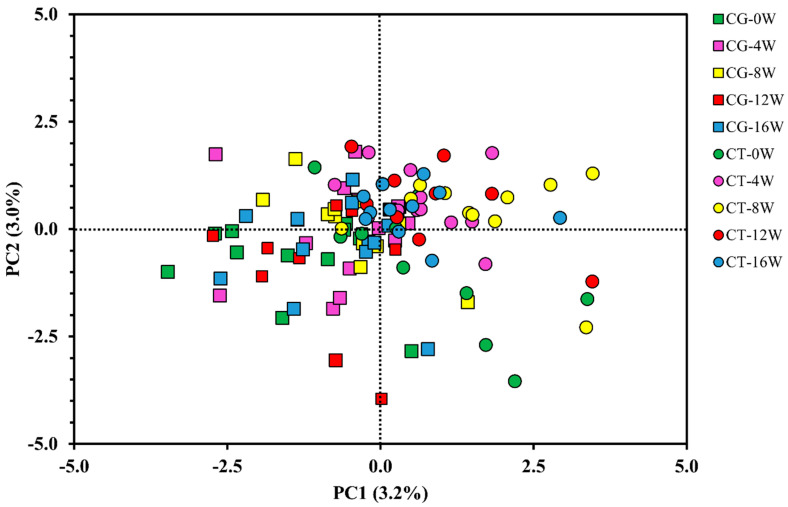
Partial least squares discriminant analysis (PLS-DA). Control Group (CG) is represented by the square (☐) and the Combined Training (CT) is represented by the circle (◯). Colours vary according to the time of data collection; 0W = green: baseline; 4W = pink: 4 weeks; 8W = yellow: after 8 weeks of training; 12W = red: after 12 weeks of training; 16W = blue: after 16 weeks of training. The present model used data for all 66 metabolites detected.

Principal component analysis (PCA) was performed to examine the differences in the overall metabolomic profile between groups and study time points. Partial least squares discriminant (PLS-DA) was performed to identify the main discriminators of the metabolic changes between groups. However, there was no segregation between the groups or validated discriminating metabolites (Figure 1 and Figure 2).

We examined the time-course of change for each of the 66 metabolites identified in the CT and CG groups and most of them did not change over time. The chemical classes of each characterized metabolite are summarized as: Organic Carbonic Acid (1), Carboxylic Acids (12), Fatty Acids (7), Amino Acids (15), Keto Acids (3), Organic Oxygen Compounds (11), Organic Nitrogen Compounds (5), Dihydrofuran (1), Hydroxy Acids (5), Imidazopyridines (2), Purine Nucleotides (1) and others with classes not shown (3) [40]. These data are shown in more detail in Supplementary Figures S1–S12. Four metabolites were found to change in the CT group (Table 3). Specifically, we noted increases in 2-Oxobutyrate at 4W (*p* = 0.012) and 8W (*p* < 0.001) and 3-Hydroxybutyrate and Ornithine at 16W (*p* = 0.022 and *p* = 0.002, respectively), with a decrease in Acetoacetate at 12W (*p* = 0.008) and 16W (*p* = 0.005). No changes were observed for the CG group (Table 3). 

In addition, some time-effects were noted for 2-Hydroxyisocaproate (*p* = 0.01), 2-Hydroxyvalerate (*p* = 0.009), Ascorbate (*p* = 0.01), Butyrate (*p* = 0.005), Creatine phosphate (*p* = 0.03), Glucose (*p* = 0.04), Isopropanol (*p* = 0.03), Methylamine (*p* = 0.01), Oxypurinol (*p* = 0.04) and Succinate (*p* = 0.03) (Supplementary Figures S2, S3 and S6–S9).

## 4. Discussion

A 16-week combined exercise training programme was able to increase cardiorespiratory fitness and muscle strength in a cohort of female participants with hypertension and MetS. However, the expected benefits to MetS components were not seen [23,43,44]. Although acute exercise has been shown to induce significant metabolic changes [45], a metabolomics analysis of serum found that only four metabolites underwent significant changes after a longer term 16-week training programme (2-oxobutyrate, 3-hydroxybutyrate, ornithine and acetoacetate). This may reflect a new settled metabolic state being established after a longer-term intervention, compared to a potential broader but transient metabolic perturbation induced acutely by exercise. 

For ornithine, the increase was not seen until 16W of CT, suggesting a need for longer-term stimulation or that it requires longer stimulation in this population, since it was increased after only 8 weeks of endurance training in a younger group of men [10]. Ornithine is a non-essential amino acid which plays a central role in the urea cycle, converting the ammonia from protein catabolism to be excreted as urea. Supplementation with L-Ornithine has been shown to attenuate fatigue during exercise by modulating lipid and amino acid metabolism [46,47] and it also improves sleep quality [48]. The increase in ornithine could be a beneficial response to exercise training, supporting protein turnover. On the other hand, it is noteworthy that the natural accumulation of intermediate metabolites from the urea cycle, such as ornithine, could represent a decreased rate of clearance of urea by the kidneys [49] and be a negative effect since it is toxic for hepatocyte mitochondria [50]. 

The training intervention also affected the production of the ketone 3-hydroxybutyrate, possibly mediated by the positive effect of extended exercise training on the gut microbiome, which leads to an increase in the butyrate-producing bacteria [51,52]. Colonocytes are another source of ketone bodies, predominantly acetoacetate and 3-hydroxybutyrate, which can freely diffuse across the plasma membrane. Both were altered with exercise training here. The reduction in acetoacetate concentration at 12W of CT was followed by an increase in 3-hydroxybutyrate at 16W, with maintenance of lower acetoacetate at this time point. Although these changes would indicate changes in a similar direction of ketoacidosis, the mean ratio of acetoacetate/3-hydroxybutyrate was constant across all time-points. The need for regulation of ketone bodies occurs when the glycogen stores in the liver are depleted and since exercise increases glycogen consumption, it is expected that ketone bodies would be affected due to an increase in fatty acid oxidation in response to exercise [53,54,55]. Vieira et al. [56] showed increases in 3-hydroxybutyrate with exercise training and this increase was associated with increased glucose metabolism, lower serum triglycerides and reduced hepatic lipid content [56], as well as improved energy production in brain and skeletal muscle [57,58]. However, there are also contradictory findings, with one in newly enlisted soldiers, showing that fatty acids and ketone body substrates were dramatically decreased in plasma in response to increased aerobic fitness after 80 days of combined training [59]. In addition, a study in overweight and obese adolescents found reduced 3-hydroxyisobutyrate after 12 weeks of a combined training intervention simultaneously with improvement in body composition [17]. Thus, it is difficult to confirm whether the changes observed in this study were due to direct effects of exercise training or body composition improvement. Here we did not see changes in body composition or dietary pattern, and the comparison with a control group reinforces the suggestion that the changes we saw are exclusively due to exercise training effects. If this effect can be confirmed, exercise would contribute to balancing gut microbiota and maintaining the mucosal barrier, which in turn modulates the host immune response, prevents infections and regulates energy expenditure [60].

The increase in 2-oxobutyrate observed here is also further evidence that exercise contributes to improved oxidative phosphorylation in these older women. The 2-oxobutyrate is one of the degradation products of threonine, which can be converted into propionyl-CoA, then methylmalonyl CoA and succinyl CoA and finally enter tricarboxylic acid (TCA) [61]. Thus, 2-oxobutyrate underwent an early increase at 4W and again at 8W, returning to baseline levels after 12W and 16W. This raises the possibility that improvements in the citric acid cycle, namely increased 2-oxobutyrate, precedes later improvement in ketone body production, and the higher 2-oxobutyrate possibly does not need to be maintained when ketone bodies become available. On the other hand, we were not able to confirm this hypothesis, since succinyl-CoA cannot be detected via NMR and also because higher 2-oxobutyrate in the blood could result in a few other fates, such as travel to the liver to supply amino acid metabolism.

Other studies testing different exercise protocols in different populations also have seen changes in TCA cycle intermediates [11]. These changes, together with the changes we observed in ketone bodies, suggest an improvement towards higher fat oxidation that would make sense with the high increments in cardiorespiratory fitness observed. The effect of exercise increasing muscle and systemic fatty acid oxidation is well known [53,54,55], but the exercise modulation of these metabolites in the blood support the hypothesis that exercise could be affecting the metabolism of other cells, such as circulating immune cells, and thus affecting their function [62,63,64,65].

Researchers have also tested the chronic effects of exercise training on resting metabolomics in different populations and they all similarly showed just a few changes in metabolites. Duft et al. [9] tested a 24-week combined training intervention in obese men and found a distinct difference in the global serum metabolomic profile between control and trained groups. Changes were seen in the levels of twenty metabolites and four were classified as best discriminators (tyrosine, 2-oxoisocaproate, histidine, pyruvate). Interestingly, the changes in some metabolites, such as glucose, tyrosine, glutamine and pyruvate, were correlated with some functional benefits, such as higher muscle strength, the VO_2_ peak and body composition improvements [9]. Compared to our findings, they also reported an increase in ornithine for the training group and the higher ornithine was associated to lower insulin. However, Silva et al. [12] also conducted a study of combined training in older adults for 36 weeks but found no differences between the intervention and control groups, suggesting variation in response dependent upon subject demographics likely to affect metabolism, such as age, BMI and the presence of syndromes such as MetS. To this end, metabolomic profiles have been shown to predict the inter-individual variability to improve cardiorespiratory fitness with aerobic training in young adults [17]. Combining the analysis of serum and muscle metabolites, Castro et al. [17] identified metabolites derived from amino acid and carbohydrate metabolism and were able to differentiate responders from non-responders to exercise.

Using different exercise regimes, Castro et al. [10], compared the effect of continuous endurance training and high-intensity interval training for 8 weeks on healthy young men and found an association of baseline serum (o-acetylcarnitine, 3-hydroxybutyrate, propyleneglycol) and skeletal muscle metabolites (alanine, glutamate, histidine, phenylalanine, proline, threonine, creatinine, glutathione, isobutyrate, 3-methylxanthine, AMP, 2-phosphoglycerate, histamine and pyruvate concentrations among others) to be indicative of amino acid and carbohydrate metabolism with cardiorespiratory fitness gain. Brennan et al. [11] also reported no difference between the effect of three different types of exercise protocols (low volume and low intensity, high volume and low intensity and high volume and high intensity) on metabolomics. In their study there were significant changes in seven metabolites after 24 weeks of different exercise protocols in middle-aged obese men and women. Tryptophan metabolites (indoxylsulfate and indole-3-lactic acid), adenosine triphosphate and malonic acid were increased; while metabolites derived from energy metabolism pathways (aconitic acid), pyruvic acid and the purine degradation product xanthine were decreased. They also found the variations in metabolite concentrations were associated with favourable responses, such as changes in waist circumference, BMI, cardiorespiratory fitness and fasting insulin levels. For example, changes in Isocitric acid, a TCA cycle intermediate was positively correlated with cardiorespiratory fitness. They also found that changes in gluconeogenic amino acids (alanine, tyrosine and proline) and the hexosamine end-product, uridine diphosphate N-acetylglucosamine (UDP-GlcNAc), were positively associated with changes in BMI and that UDP-GlcNAc was associated with changes in waist circumference and insulin metabolism [11]. 

### Limitations and Future Perspectives

Our exploratory analyses were based on only one metabolomic technique and a relatively small number of metabolites were detected. Therefore, other complementary measures, such as mass spectrometry [66], can help to identify serum metabolites undetected in this study [67,68].

We believe that some of the differences between our study and others could also be due to our study being the first to investigate exercise training effects on metabolomics in older women with hypertension and MetS, as individuals with different cardiometabolic traits undergo a wide range of exercise training responsiveness [69]. Nevertheless, it is noteworthy that most studies testing chronic exercise training effects on metabolomics used aerobic training rather than CT. The different effects seen for both types of training on human metabolic profile needs further research. While most of the physical fitness and metabolic benefits can be achieved by CT in older adults with hypertension, some benefits are only obtained by aerobic training [70], and aerobic training can lead to more health benefits than CT in individuals with MetS [8].

It is important to add that, although these older women had hypertension and MetS, their baseline lipid, glycaemic and inflammatory profile were within the normal range and their blood pressure was maintained with medication, which may have contributed to the modest changes in their metabolic profile. This is in agreement with the small or null exercise training effects observed for a variety of cardiometabolic health markers explored in the analysis of the full cohort from which this sub-study was derived [23].

Although this study analysed a small sample, we highlight the challenge in conducting lengthy exercise interventions in humans under a rigorous monitoring regimen. It is noteworthy, since we observed a very low magnitude of effect (Cohen’s d) for most metabolites, a higher sample size would probably not reveal many more changes. As far as we know, only a few studies have explored the adaptations promoted by combined training from a metabolomic perspective [9,71,72], but none of them have included participants with metabolic syndrome. However, since we noticed a sequential number of changes in 2-oxobutyrate, 3-hydroxybutyrate, acetoacetate and ornithine, we encourage future studies to investigate the influence of CT on ketone body production, the citric acid cycle and the urea cycle, as well as its relationship with oxidative phosphorylation and exercise fatigue.

## 5. Conclusions

The analysis of the time-course effects of CT on the metabolomic profile in older women with MetS and hypertension led us to identify potential flux through the citric acid cycle (increase in 2-oxobutyrate), followed by the improved production of ketone bodies (3-hydroxybutyrate) that together might explain the known improvement in fatty acids oxidation with exercise. The meaning of the latter increases in ornithine with CT is still unclear. We encourage future studies to identify the association between these metabolic pathways and clinical outcomes in this population.

## Figures and Tables

**Table 1:** Pre- and post-training characteristics of participants.

	CG (*n* = 13)		CT (*n* = 12)	
Variables	0W	16W	0W	16W
Age (years)	65.6 ± 5.6	-	64.5 ± 3.9	-
Body mass (kg)	75.9 ± 8.3	76 ± 8.7	80.7 ± 6.3	80.3 ± 7.1
BMI (kg∙m^−2^)	30.5 ± 2.6	30 ± 2.7	30.3 ± 2.3	30.1 ± 2.5
Body fat (%)	46.3 ± 4.4	47.2 ± 4.4	45.3 ± 3.5	45.1 ± 3.2
Waist circumference (cm)	103.2 ± 4.8	103.2 ± 4.4	106.3 ± 5.7	103.2 ± 6.5
Systolic BP (mm Hg)	132.1 ± 27.9	125.5 ± 20.1	133.8 ± 21.3	126 ± 11.9
Diastolic BP (mm Hg)	84.9 ± 18	78.1 ± 9.2	84.6 ± 10.8	82.5 ± 9.1
Glycemia (mg∙dL^−1^)	101.9 ± 13.3	97.4 ± 9.9	109.7 ± 27.7	109.1 ± 25.8
Triglycerides (mg∙dL^−1^)	127.2 ± 48.8	123.1 ± 48.1	105.7 ± 45.9	124.5 ± 47.1
Total cholesterol (mg∙dL^−1^)	170.8 ± 47.0	173.9 ± 50.4	174.6 ± 39.1	178.2 ± 46.8
HDL-cholesterol (mg∙dL^−1^)	41.0 ± 11.6	42.5 ± 15.2	41.2 ± 12.1	43.6 ± 21.6
LDL-cholesterol (mg∙dL^−1^)	105.2 ± 38.1	106.7 ± 39.1	112.2 ± 34.0	109.7 ± 32.9
CRP (mg∙L^−1^)	2 ± 0.4	1.5 ± 0.6	1.8 ± 0.5	1.3 ± 0.3
TNF-α (pg∙mL^−1^)	1.4 ± 0.2	1.6 ± 0.2	1.5 ± 0.1	1.8 ± 0.3

Data are presented as mean ± standard deviation. CG = Control Group; CT = Combined Training; CRP = C- reacive protein; TNF-α = tumour necrosis factor alpha; HDL = high density lipoprotein; LDL = low density lipoprotein; 0W = baseline; 16W = post 16 weeks.

**Table 2:** Resistance training load progression in the CT group.

RT Load (kg)	W0	W4	W8	W12	W16
Leg extension	11.73 ± 2.2	17.73 ± 3.9 ^a^	21 ± 4.9 ^a^	25.45 ± 4.7 ^ab^	27.18 ± 5.1 ^abc^
Leg flexion	11.73 ± 3	16.64 ± 2.9	20.55 ± 5.4 ^a^	22.27 ± 4.4 ^ab^	22.64 ± 5.1 ^ab^
Leg press	23.64 ± 10.3	37.27 ± 7.9	50 ± 12.6 ^a^	61.82 ± 10.8 ^ab^	64.55 ± 11.3 ^abc^
Bench press	3.18 ± 1.3	4.64 ± 1.4	6.09 ± 1.2 ^a^	6.45 ± 1.4 ^a^	6.64 ± 1.4 ^ab^
Lat pulldown	15.45 ± 2.7	19.09 ± 3.8	20.91 ± 4.4 ^a^	22.27 ± 4.1 ^a^	23.64 ± 4.5 ^a^

Data are presented as mean ± standard deviation. RT = Resistance Training; ^a^ = significantly different from 0W (*p* ≤ 0.05); ^b^ = significantly different from 4W (*p* ≤ 0.05); ^c^ = significantly different from 8W (*p* ≤ 0.05).

**Table 3:** Metabolites that showed significant differences in a linear mixed model analysis.

**Metabolite**	**CT (mM)**				
	**0W**	**4W**	**8W**	**12W**	**16W**
2-Oxobutyrate	0.0029 ± 0.0009	0.0038 ± 0.0011 ^a^	0.0041 ± 0.0015 ^a^	0.0038 ± 0.0012	0.0034 ± 0.0011
3-Hydroxybutyrate	0.0717 ± 0.0377	0.0483 ± 0.0421	0.0420 ± 0.0219	0.0372 ± 0.0186	0.0397 ± 0.0331 ^a^
Acetoacetate	0.0441 ± 0.0240	0.0299 ± 0.0214	0.0268 ± 0.0127	0.0224 ± 0.0071 ^a^	0.0239 ± 0.0141 ^a^
Ornithine	0.0100 ± 0.0028	0.0110 ± 0.0025	0.0098 ± 0.0036	0.0105 ± 0.0030	0.0182 ± 0.0152 ^b^
	**CG (mM)**				
	**0W**	**4W**	**8W**	**12W**	**16W**
2-Oxobutyrate	0.0026 ± 0.0009	0.0026 ± 0.0010	0.0028 ± 0.0007	0.0026 ± 0.0009	0.0032 ± 0.0012
3-Hydroxybutyrate	0.0437 ± 0.0384	0.0275 ± 0.0111	0.0387 ± 0.0317	0.0475 ± 0.0598	0.0523 ± 0.0361
Acetoacetate	0.0260 ± 0.0188	0.0196 ± 0.0068	0.0220 ± 0.0148	0.0261 ± 0.0260	0.0277 ± 0.0136
Ornithine	0.0116 ± 0.0050	0.0110 ± 0.0047	0.0104 ± 0.0038	0.0117 ± 0.0046	0.0102 ± 0.0041

Data are presented as mean ± standard deviation. CG = Control Group; CT = Combined training group; 0W = baseline; 4W = 4 weeks; 8W = 8 weeks; 12W = 12 weeks; 16W = 16 weeks; ^a^ = significantly different from 0W (*p* ≤ 0.05); ^b^ = significantly different from 0W, 4W, 8W and 12W (*p* ≤ 0.0.5).

## Data Availability

The data presented in this study are available on request from the corresponding author. Data is not publicly available due to privacy.

## Supplementary Materials

The following supporting information can be downloaded at: https://www.mdpi.com/article/10.3390/metabo12111041/s1, Figure S1: Class of identified organic carbonic acid and its concentration in mM; Figure S2: Class of identified carboxylic acids and their concentrations in mM; Figure S3; Class of identified fatty acids and their concentrations in mM; Figure S4: Class of identified amino acids and their concentrations in mM; Figure S5: Class of identified keto acids and their concentrations in mM; Figure S6: Class of identified organic oxygen compounds and their concentrations in mM; Figure S7: Class of identified organic nitrogen compounds and their concentrations in mM; Figure S8: Class of identified dihydrofuran and its concentrations in mM; Figure S9: Class of identified hydroxy acids and their concentrations in mM; Figure S10: Class of identified imidazopyridines and their concentrations in mM; Figure S11: Class of identified purine nucleotides and their concentrations in mM; Figure S12: Classes not shown, identified and their concentrations in mM.
