# Supplementary material for: Metabolomic Response throughout 16 Weeks of Combined Aerobic and Resistance Exercise Training in Older Women with Metabolic Syndrome
Source: Metabolites. 2022 Oct 30;12(11):1041. doi: 10.3390/metabo12111041 (PMC9693245; doi:10.3390/metabo12111041)
Supplement: Supplementary file 1 [file metabolites-12-01041-s001.zip › metabolites-1982109-supplementary.pdf]

Supplementary material

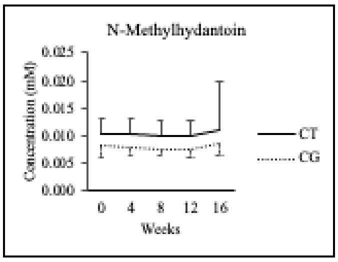

Figure S1. Class of identified organic carbonic acid and its concentration in mM.

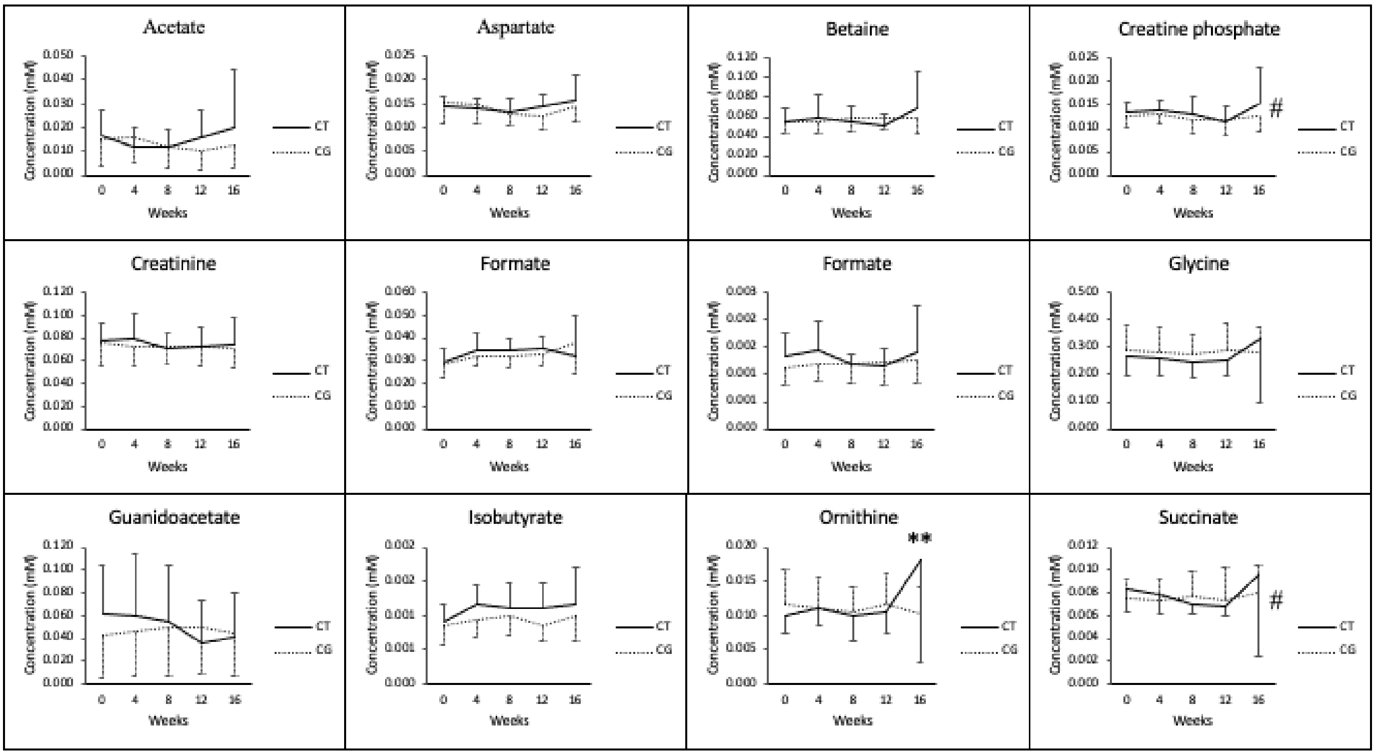

Figure S2. Class of identified carboxylic acids and their concentrations in mM. ** significantly different from 0W, 4W, 8W and 12W within CT ($P \leq 0.05$); #: significant time-effect ($P \leq 0.05$).

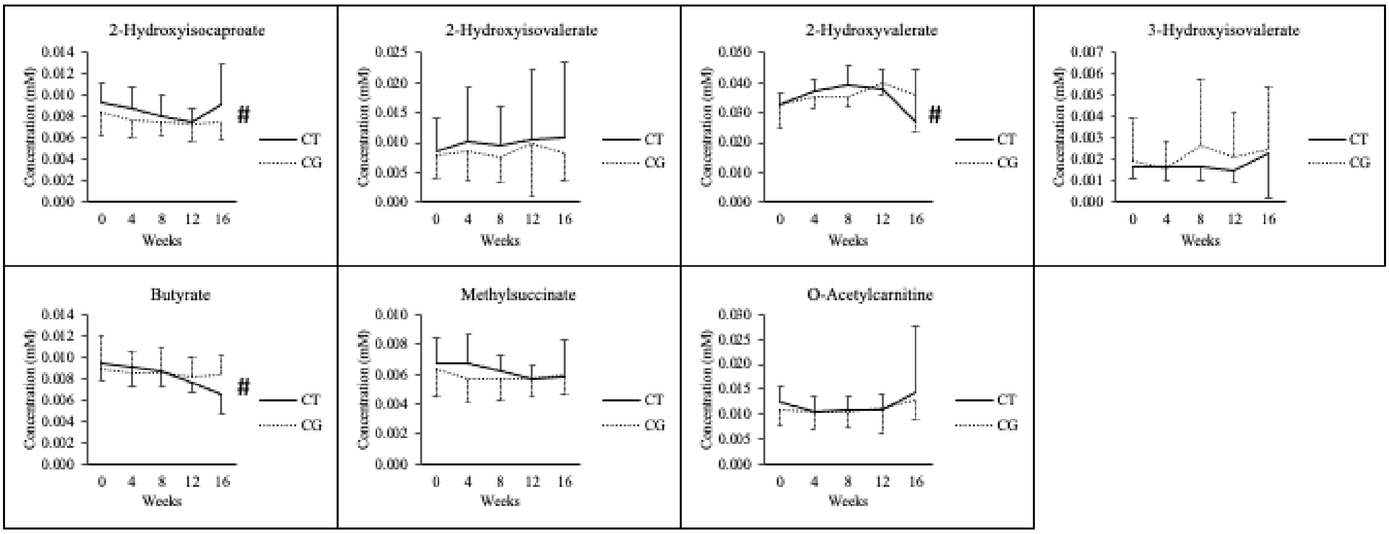

Figure S3. Class of identified fatty acids and their concentrations in mM. #: significant time-effect ($P \leq 0.05$).

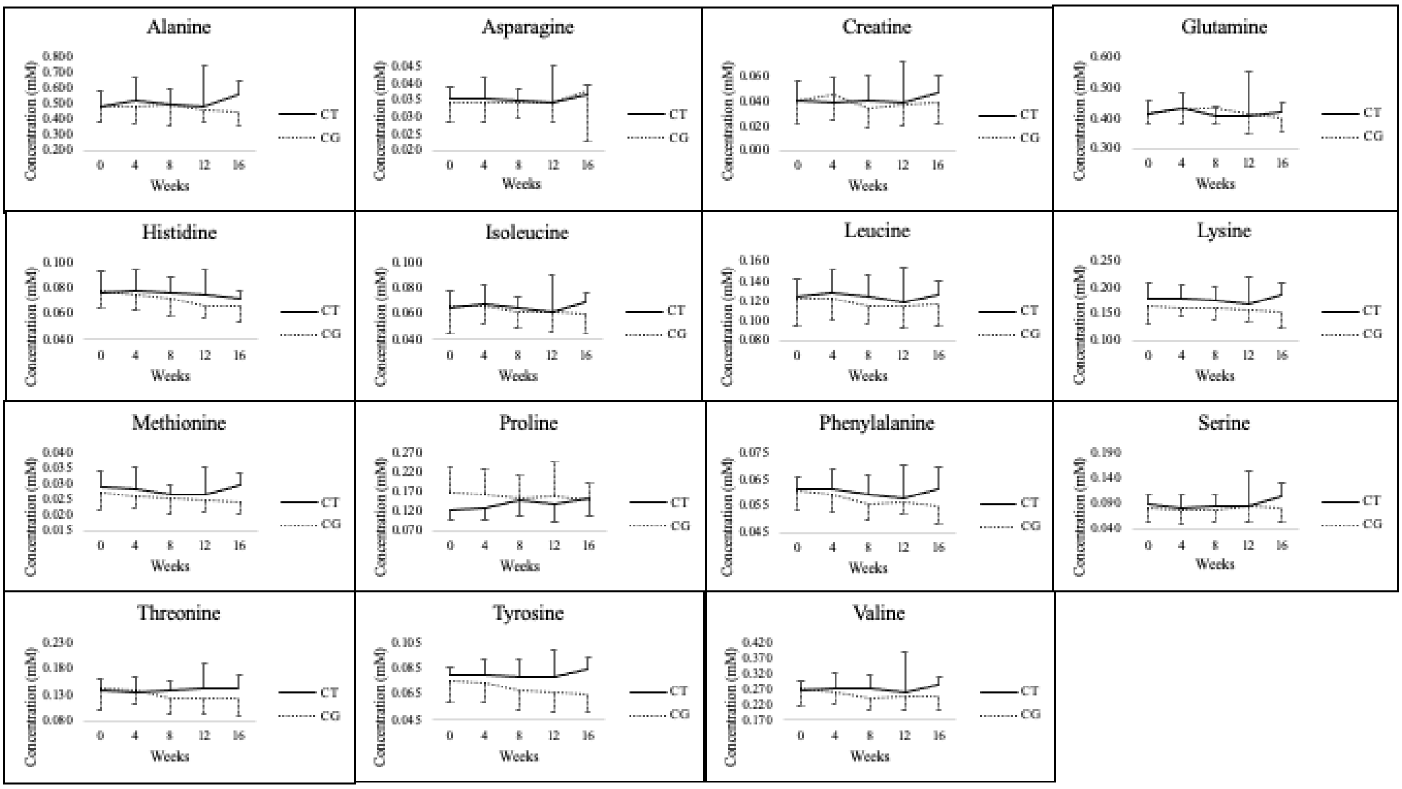

Figure S4. Class of identified amino acids and their concentrations in mM.

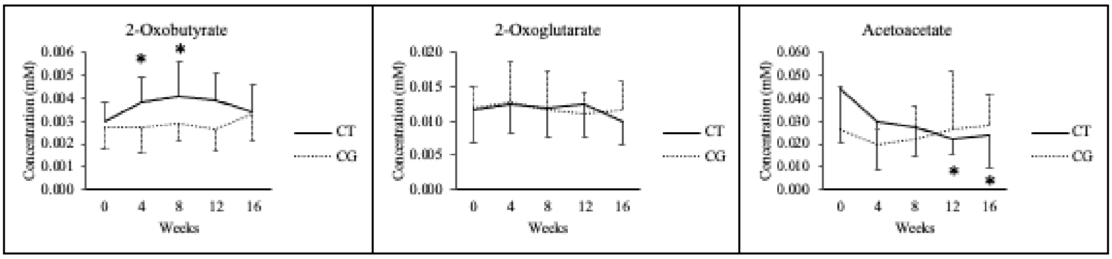

Figure S5. Class of identified keto acids and their concentrations in mM. *: significantly different from 0W within CT ($P \leq 0.05$).

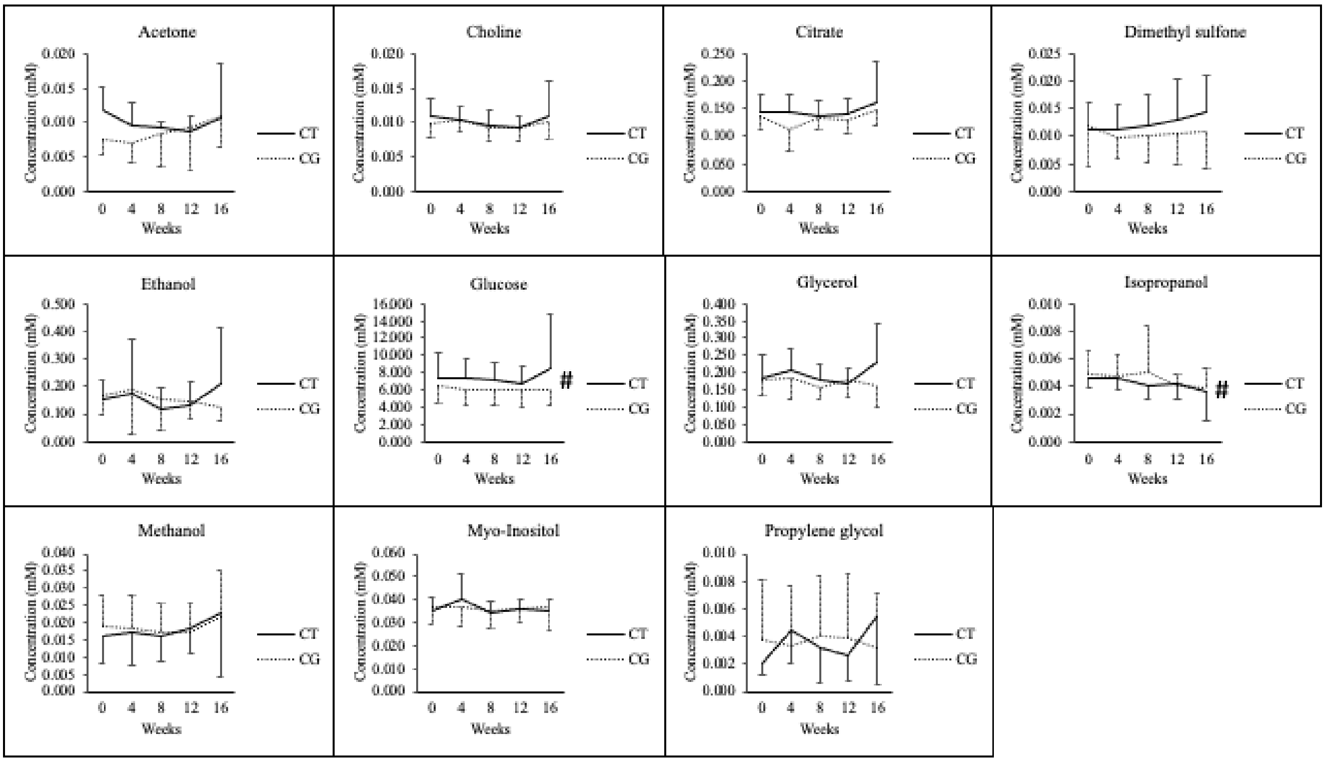

Figure S6. Class of identified organic oxygen compounds and their concentrations in mM. #: significant time-effect ($P \leq 0.05$).

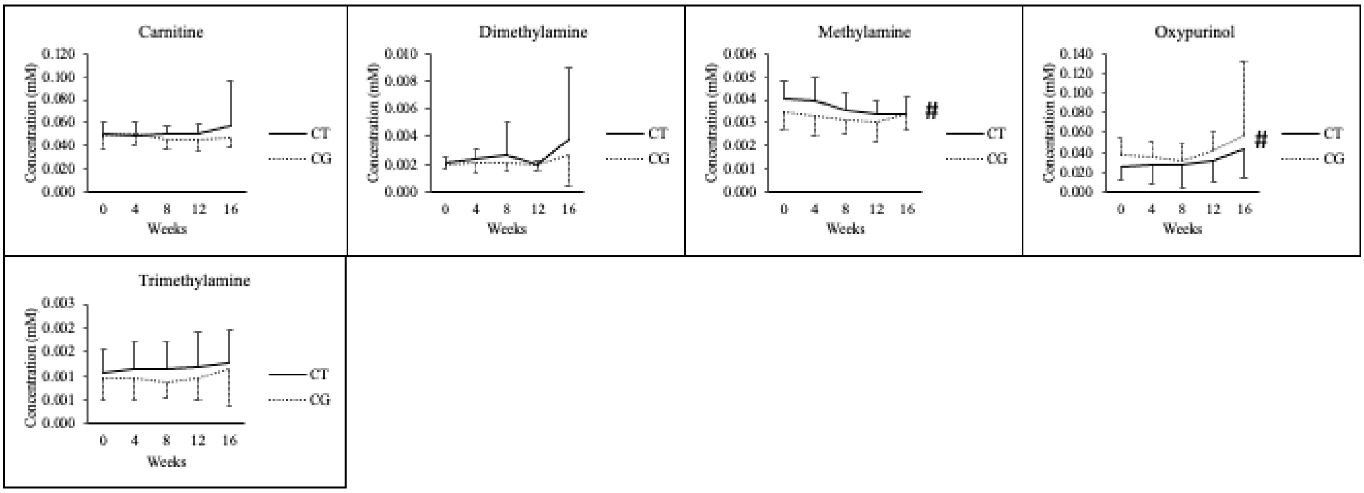

Figure S7. Class of identified organic nitrogen compounds and their concentrations in mM. #: significant time-effect ($P \leq 0.05$).

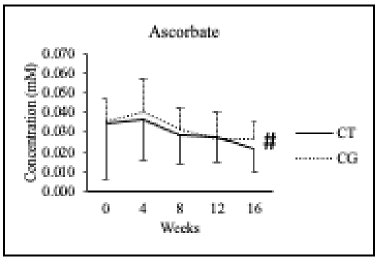

Figure S8. Class of identified dihydrofuran and its concentrations in mM. #: significant time-effect ($P \leq 0.05$).

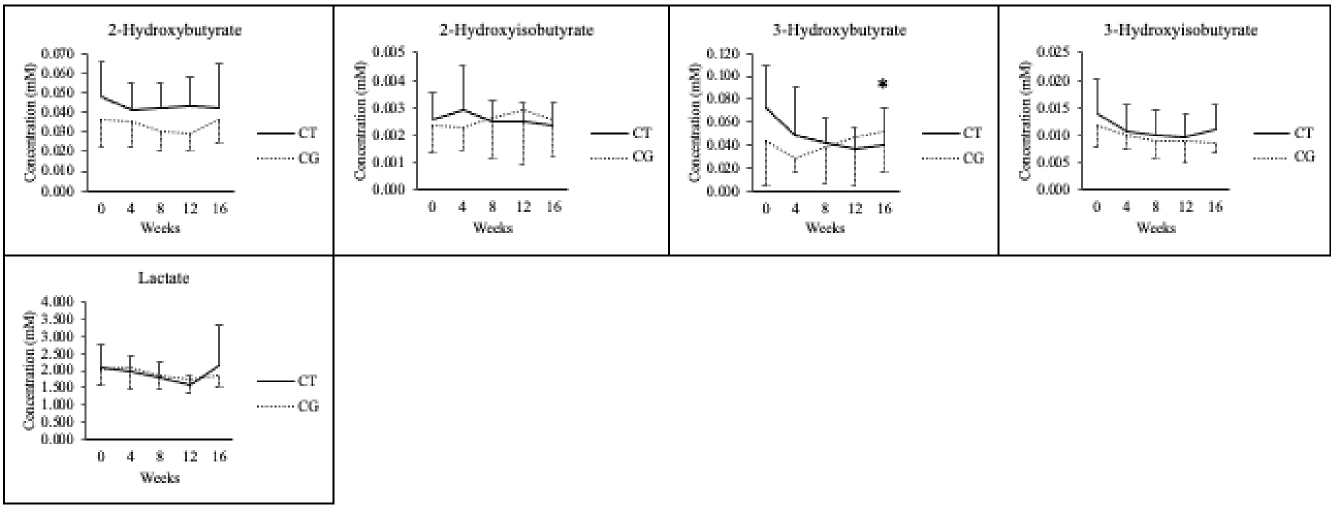

Figure S9. Class of identified hydroxy acids and their concentrations in mM. *: significantly different from 0W within CT ($P \leq 0.05$).

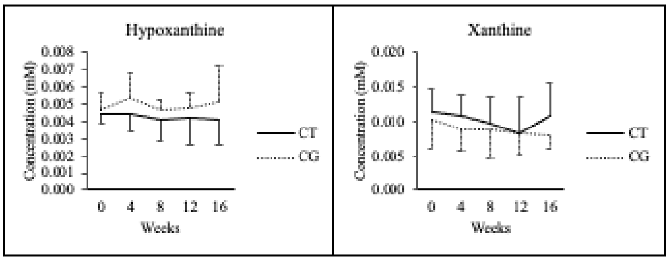

Figure S10. Class of identified imidazopyridines and their concentrations in mM.

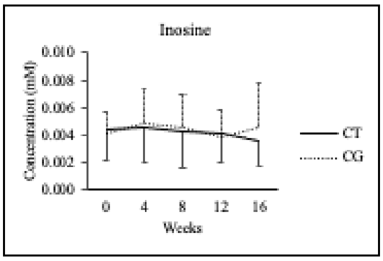

Figure S11. Class of identified purine nucleotides and their concentrations in mM.

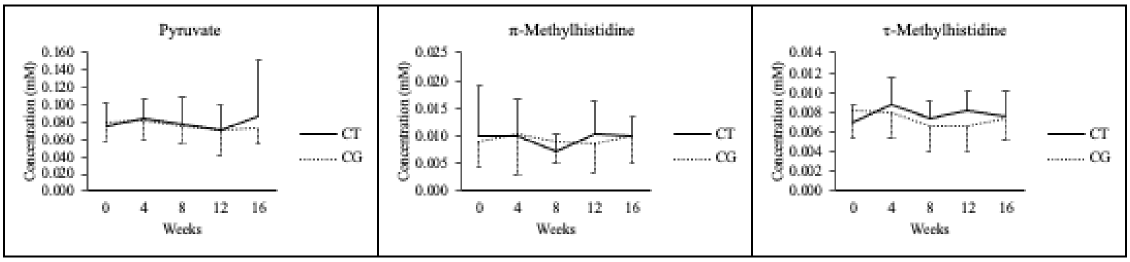

Figure S12. Classes not shown, identified and their concentrations in mM.
